# Use of maraviroc in patients with undetectable viral load: efficacy, tolerance and predictors of viral response in MARAVIROC-cohort study

**DOI:** 10.7448/IAS.17.4.19800

**Published:** 2014-11-02

**Authors:** María Jesús Pérez Elías, David Arroyo, Alberto Diaz, Cristina Herrero, Loreto Martinez-Dueñas, Ana Moreno, Jose Hernández-Quero, Daniel Podzamcer, Cristina Gomez-Ayerbe, Jose Luis Casado, Javier Zamora, Antonio Rivero, Santiago Moreno, Josep María Llibre

**Affiliations:** 1 Infectious Diseases, Hospital Ramón y Cajal, IRYCIS, Madrid, Spain; 2 Statistics Department, Hospital Ramón y Cajal, IRYCIS, Madrid, Spain; 3 FLS-Research Support, Hospital Germans Trias i Pujol, Barcelona, Spain; 4 Infectious Diseases, Hospital Universitario Reina Sofía, Córdoba, Spain; 5 Infectious Diseases, Hospital San Cecilio, Madrid, Spain; 6 Infectious Diseases, Hospital de Bellvitge, Barcelona, Spain; 7 Infectious Diseases, Hospital Universitario Reina Sofía, Córdoba, Spain; 8 Infectious Diseases, Hospital Germans Trias i Pujol, Barcelona, Spain

## Abstract

**Introduction:**

No controlled clinical trials had studied the role of maraviroc (MRV) in fully suppressed patients [1].

**Materials and Methods:**

MRV-cohort is an observational, retrospective, multicentric (27 sites) large cohort study of patients starting MRV in clinical practice under different circumstances, with at least 48 weeks of follow-up. For the present analysis we selected all those patients starting with an HIV-RNA<50 copies/mL. Demographics, baseline CD4 cell count, past history of antiretroviral treatment (ART), tropism, reasons for MRV use, MRV based therapy and change/end of MRV use were assessed. Paired analysis of lipid, hepatic and kidney profile changes and univariate and multivariate analyses of HIV-RNA<50 copies/mL at 48 weeks were explored.

**Results:**

We included 247 out of 667 subjects from the entire cohort. At study entry, their median age was 47 years, 23% were women, 31% MSM, 49% had CDC category C, median CD4+ counts were 468 cells/mm^3^, 46% were HCV+ and 4.5% AgHBs+. Tropism information was available in 197 (94% R5). Median length of prior ARTV was 10.7 years, with exposure to a median of three drug families. Main reasons for prescribing MRV were: toxicity 38%, inmunodiscordance 23%, simplification 19% and admission in a clinical trial 10.4%. MRV based therapies used were MRV+2NRTIs 9%, MRV+PI 46%, MRV+PI+other 40% and MRV+other 5%. At 48 weeks, 23% of patients had changed or finished MRV therapy due to toxicity 2.4%, virological failure 2%, immunological failure 1.2%, simplification 3,2%, trial requirement 9.7%, medical decision 2.8%, treatment suspension 1.2% and unknown 0.4%. At 48 weeks, no significant changes were observed in lipid, hepatic or kidney profiles, and 85% of patients remained with HIV-RNA<50 copies/mL. Focusing on viral response univariate and multivariate models did not show any significant baseline variable explaining viral failure.

**Conclusions:**

In clinical practice MRV was used, mostly in R5 positive patients, with adequate efficacy and tolerance, but important number of patients changed due to non-clinical reasons. In this scenario neither reason for use of MRV nor MRV-based therapy explained viral failure.
